# Supplementary material for: Drivers and dynamic mechanisms of sports tourism integration in cross-border regions: Evidence from the Guangdong-Hong Kong-Macao Greater Bay Area
Source: PLoS One. 2026 Mar 23;21(3):e0344124. doi: 10.1371/journal.pone.0344124 (PMC13008054; doi:10.1371/journal.pone.0344124)
Supplement: S1 Table — (DOCX) [file pone.0344124.s001.docx]

**S1 Table. Example of open coding.**

| **Initial Category** | **Raw Data (Initial Concept)** | **References** |
| --- | --- | --- |
| **A1 Industry Policy** | The government has issued relevant policies to encourage the integrated development of sports events and tourism industry, which has pointed out the direction for the planning and holding of our events (a1 policy support).; The government has included the "China Cup Sailing Regatta" organized by our enterprise as a key international event in Shenzhen, providing comprehensive guidance and support (a2 Government Guidance) | Zhong Y. & Xu Y. [24], Ye C.[25] |
| **A2 Fiscal Policy** | The government supports our enterprise in hosting sports events through measures like establishing a special fund for sports industry development (a3 Financial Support) and offering event subsidies (a4 Fiscal Subsidy), reducing the financial burden on the enterprise. | Cao K. et al. [21], Wang S. et al. [26] |
| **A3 Financial Policy** | The government has implemented policies for subsidized loans for sports enterprises (a5 Financing Policy), guiding financial institutions to provide loans with subsidies and preferential interest rates (a6 Financial Support), which lowers the operating costs for the enterprise. | Wang Y. et al. [18], Ma Y. & Li H. [27] |
| **A4 Resource Endowment** | We leverage the abundant outdoor sports tourism resources in Guangzhou (a7 Sports Tourism Resources) to design a series of popular outdoor sports projects; we choose destinations with well-developed sports facilities and tourism services (a8 Infrastructure) to ensure a safe, comfortable, and enjoyable experience for tourists; Guangzhou, as a transportation hub city, offers convenient transportation that allows tourists to easily reach and participate in our outdoor sports tourism projects (a9 Transportation Location). | Zeng Y. & Zhang X. [19], Lyu, S [28] |
| **A5 Innovation Environment** | We take full advantage of the innovation atmosphere in GBA, continuously launching new sports tourism products and services, such as "Hot Snow Miracle" and "Sunac Sports World," which combine ice and snow sports with sports entertainment, attracting a large number of tourists (a10 Innovation Atmosphere); we actively seek cross-industry collaborations with live broadcasting, entertainment, and event industries to promote the innovative development of sports tourism (a11 Cross-industry Cooperation). | Liu X. [29], Lei B. [30] |
| **A6 Enterprise Performance** | Sports tourism products usually have high added value and profit margins, such as equipment rentals and coach training services, which help us achieve higher revenue by expanding the sports tourism business (a12 Enterprise Profit); As a new form of tourism, sports tourism attracts a large number of young tourists seeking novelty and excitement, and expanding into this market helps us increase our market share (a13 Market Expansion). | Jin Y. et al. [31], Chen W. [32] |
| **A7 Industry Upgrade** | By integrating sports and tourism resources, we offer a diversified range of sports tourism products such as sports viewing tours and outdoor sports experience tours, helping us transition from a traditional tourism product provider to a comprehensive tourism service provider (a14 Industry Transformation and Upgrade); Through industry chain extension, we can control more resources, enhance our bargaining power, and provide a richer sports tourism experience for tourists (a15 Industry Chain Extension). | Yang Q.[33], Lu Z. et al. [34] |
| **A8 Consumer Demand** | International sports events such as the Olympics and European Cup have a huge impact on consumer travel demand, with bookings for group tours to France during the Paris Olympics rising by 225% (a16 Demand Potential); To meet consumers' diverse demands for sports tourism products, we constantly innovate and expand our product lines, such as developing sports viewing tours, hiking, cycling, water sports, and other sports tourism products, to meet the needs of different consumers (a17 Demand Diversification). Tourists' demand for experiential travel continues to rise, with more emphasis on participatory, interactive, and personalized experiences, prompting us to break the traditional boundaries between tourism resources and sports, achieving deeper integration (a18 Demand Content Changes). | He S. et al. [20], Zarei A .[35] |
| **A9 Market Competition** | In the fierce market competition, we realize that only continuous innovation can maintain a competitive edge, so we actively explore the intersection of sports and tourism, launching a series of innovative sports tourism products (a19 Competition Drive); Market competition pushes us to continuously improve service quality, focusing on details and experience in all aspects of sports tourism product design, sales, and execution, ensuring that tourists receive professional and thoughtful service (a20 Service Quality Improvement); To attract more customers (a21 Market Expansion), we analyze consumer needs and market trends, launching esports tourism products and fitness tourism products. | Ratten V.[10]  Tassiopoulos D.[36] |
| **A10 Technology Application** | We introduced an intelligent event management system to automate processes such as event registration, check-in, and result posting, improving event organization efficiency and accuracy, and enhancing the satisfaction and loyalty of event tourists (a22 Resource Optimization); Through technology platforms, sports and tourism enterprises can easily exchange information and share resources, jointly developing new products and services (a23 Industry Collaboration). | Mao X. & Li T. [11], Hou Y. et al. [37] |
| **A11 Innovation Driven** | We use big data to analyze tourist behavior and preferences, providing more personalized sports tourism routes and sports event recommendations (a24 Service Innovation); In Sunac Sports World in Guangzhou, we have introduced technologies such as virtual reality and augmented reality to provide tourists with a more immersive sports experience (a25 Product Innovation); Our enterprise has developed sports tourism apps, e-commerce platforms, and other new business models, further expanding the sports tourism market (a26 Market Innovation). | Bröring S. & Leker J.[38] |
| **A12 Innovative Talent** | Our team proposed combining outdoor research camps with sports, designing a series of sports-themed research tourism products that integrate sports training, outdoor adventure, and cultural experiences, providing personalized service options for tourists of different ages and interests (a27 Creativity); The team's creativity is reflected not only in product design but also in marketing, customer service, and operational management. These innovative ideas and practices have enabled our enterprise to stand out in the competitive market (a28 Innovative Thinking). | Tsekouropoulos G. et al. [39], Zhang S. et al. [40] |
| **A13 Employee Awareness** | Our enterprise employees have a high level of awareness regarding the sports tourism industry integration, and in the economically dynamic and culturally diverse GBA, they understand that the integration of sports and tourism is an inevitable trend (a29 Employee Awareness); They need to have knowledge in both sports and tourism sectors, understand the development trends and market needs of both industries, and find points of integration and innovation (a30 Employee Competency). | Lu Z. et al. [41], Papaioannou A. et al. [42] |
