# Supplementary material for: Drivers and dynamic mechanisms of sports tourism integration in cross-border regions: Evidence from the Guangdong-Hong Kong-Macao Greater Bay Area
Source: PLoS One. 2026 Mar 23;21(3):e0344124. doi: 10.1371/journal.pone.0344124 (PMC13008054; doi:10.1371/journal.pone.0344124)
Supplement: S2 Table — (DOCX) [file pone.0344124.s002.docx]

**S2 Table. Results of axial coding.**

| **Core Category** | **Sub-category** | **Relationship Meaning** |
| --- | --- | --- |
| Government Behavior  (GA) | A1 Industry Policy | Industry policies provide directional guidance and resource support for industry integration. |
|  | A2 Fiscal Policy | Fiscal policies provide funding support, subsidies, and other incentives, driving industry integration. |
|  | A3 Financial Policy | Financial policies provide capital support and financing convenience, ensuring funding for industry integration. |
| Resource Environment (RE) | A4 Resource Endowment | Resource endowment, including natural resources, cultural resources, and social resources, provides the initial impetus for sports tourism industry integration. |
|  | A5 Innovation Environment | The innovation environment stimulates creativity, promotes cross-industry collaboration, and drives business model innovation, which is the soft power of industry integration. |
| Enterprise Development (ED) | A6 Enterprise Performance | Enterprise performance reflects resource integration and efficiency, serving as a key driving force for the sports tourism industry integration. |
|  | A7 Industry Upgrade | The need for enterprise industry upgrade drives deep integration in product innovation, resource integration, and market expansion within the sports tourism industry. |
| Market Demand  (MD) | A8 Consumer Demand | Consumption upgrades and diversified consumer demand drive innovation in sports tourism industry integration. |
|  | A9 Market Competition | Market competition requires industry integration to continuously improve service quality, optimize resource allocation, innovate products, and meet diverse consumer needs, strengthening core competitiveness. |
| Technological Innovation  (TI) | A10 Technology Application | Technology applications promote product experience improvements, innovation upgrades, and facilitate the sharing of sports and tourism resources, creating synergies and complementing advantages. |
|  | A11 Innovation-Driven | Innovation-driven forces foster product, service, and market innovations, creating new business models and promoting deep integration of the sports tourism industry. |
| Human Capital (HC) | A12 Innovative Talent | Innovative talent, with creative thinking, creativity, and cross-disciplinary knowledge, is key to driving industry integration. |
|  | A13 Employee Awareness | Employee awareness and competency provide intellectual support and decision-making foundation for industry integration. |
