# Supplementary material for: Drivers and dynamic mechanisms of sports tourism integration in cross-border regions: Evidence from the Guangdong-Hong Kong-Macao Greater Bay Area
Source: PLoS One. 2026 Mar 23;21(3):e0344124. doi: 10.1371/journal.pone.0344124 (PMC13008054; doi:10.1371/journal.pone.0344124)
Supplement: Attachment — Submitted filename: Response to Reviewers.docx [file pone.0344124.s003.docx]

Dear editor and reviewer:

Thank you for your comments and suggestion concerning our manuscript. The comments and suggestions are all valuable and very helpful for revising and improving our paper as well as the important guiding significance to our researches. We have studied comments carefully and have made correction which we hope meet with approval.

To facilitate this discussion, we first retype your comments in italic font and then present our responses to the comments.

***Comment 1:***

*The study’s core contribution mirrors already established frameworks such as ESP (Environment-System-Policy), which has been previously applied in various regional industrial analyses. The claims of novelty are overstated, particularly as most identified drivers (government policy, resource endowment, enterprise behavior, etc.) are well-documented in existing literature.*

**Response 1:**

Thank you for your valuable suggestion regarding our research framework. We understand your concerns about the novelty of the ESP framework. In light of your feedback, we have decided to remove the ESP framework and directly identify the six key driving factors using Grounded Theory (GT), while also constructing a dynamic mechanism model. The use of Grounded Theory ensures that the research conclusions emerge naturally from the data, enhancing the scientific rigor and robustness of the study. The specific modifications are as follows:

(1) Adjustment of the Theoretical Framework: The introduction of the ESP framework has been removed, and we now directly employ Grounded Theory as the research methodology. We emphasize the scientific and rigorous nature of Grounded Theory in extracting core driving factors from the data.
(2) Reconstruction of the Theoretical Model: Using the three stages of coding in Grounded Theory (open coding, axial coding, and selective coding), we have built relationships among the driving factors. The focus is on revealing how the six driving factors (government behavior, resource environment, enterprise development, market demand, technological innovation, and human capital) interact to promote the integration of the sports tourism industry.
(3) Enhanced Empirical Analysis: We use Structural Equation Modeling (SEM) to verify the effects of the six driving factors on the outcomes of industry integration.
(4) Revised Contribution Statement: We have revised the contribution section to highlight：Firstly, the identification of the six driving factors and their dynamic mechanisms through Grounded Theory and empirical research. Secondly, the provision of specific policy recommendations for cross-border regions, such as the Greater Bay Area (GBA). Thirdly, the study addresses the gaps in existing research regarding the micro-level factors (enterprise behavior, technological innovation, and human capital).

The above revisions can be found in the sections of “Research design” “Selective coding” “Model construction” and “Discussion” in the text, with changes highlighted in red. We believe these adjustments will strengthen the clarity and focus of the study, and we hope they meet your expectations.

***Comment 2:***

*The theoretical application of ESP is not critically developed. The authors claim it integrates “environmental, systemic, and policy” dimensions but fail to operationalize these within the SEM model in a robust way.*

**Response 2:**

Thank you for your valuable feedback on this study. We fully understand your concern regarding the critical development of the theoretical application of the ESP framework. You pointed out that while the framework claims to integrate “environmental, systemic, and policy” dimensions, it fails to operationalize these within the Structural Equation Modeling (SEM) in a robust manner. In light of your feedback, we have decided to remove the ESP framework and instead directly identify the six key driving factors using Grounded Theory, while also constructing a dynamic mechanism model. This modification refines the focus of the study to data-driven conclusions, enhancing the scientific rigor and robustness of the research.

Additionally, we have employed Structural Equation Modeling (SEM) to validate the impact of the six driving factors on the effectiveness of sports tourism industry integration. The results show that all path coefficients are significant (P < 0.05), indicating that the six driving factors have a positive influence on industry integration outcomes. Specifically, Human Capital (HC) and Technological Innovation (TI) have the most significant impact on industry integration, with standardized path coefficients of 0.619 and 0.541, respectively. This finding underscores the important role of micro-level factors in driving industry integration.

The above revisions can be found in the sections of “Model construction” and “Hypothesis testing” in the text, with changes highlighted in red. We hope this addresses your concern and enhances the clarity of our theoretical approach and empirical analysis.

***Comment 3:***

*The model is visually cluttered, with arrows lacking directionality clarity and undefined pathways. No numerical values or standardized paths are shown on the figure itself.*

**Response 3:**

Thank you for your valuable feedback on the visual presentation of the model. We appreciate your observations regarding the cluttered appearance and lack of clarity in the directional arrows and pathways. In response to your concerns, we have made the following adjustments:

(1) Clarified Directionality and Pathways: We have revised the model to ensure that all arrows are clearly defined and point in the appropriate direction, improving the readability and interpretability of the diagram.
(2) Simplified Visual Layout: The overall layout of the model has been streamlined to reduce visual clutter. We have reorganized the elements in a more structured and straightforward manner to enhance clarity.
(3) Clarification on Standardized Path Coefficients: Since the path coefficients are derived from the empirical analysis (using Structural Equation Modeling, SEM), they are based on data that will be presented later in the study. As the empirical analysis has not yet been conducted at the model construction stage, we believe it is more appropriate to focus on the theoretical relationships between the variables in this diagram. We present the numerical values and standardized path coefficients after the SEM analysis in the results section, ensuring the data-driven nature of the findings, as shown in Figure 3.
(4) Updated Figure: The updated version of the Figure 2 now visually reflects the theoretical model’s structure, showing the relationships between the key factors without prematurely including the standardized path coefficients. This change improves the clarity and correctness of the figure.

The above revisions can be found in the sections of “Model construction” and “Hypothesis testing” in the text, with changes highlighted in red. We hope these revisions meet your expectations and improve the presentation of our study.

***Comment 4:***

*The claim of using grounded theory followed by SEM is methodologically inconsistent. Grounded theory typically builds theory from data without preconceived models, while SEM tests hypothesized models. This hybrid approach is not properly justified, and the transition between qualitative coding and quantitative model testing lacks transparency.*

**Response 4:**

Thank you for your insightful comments regarding the methodological approach used in our study. We understand your concern about the combination of Grounded Theory and Structural Equation Modeling (SEM), and we appreciate your suggestion to clarify the transition between these two methodologies.

You correctly pointed out that Grounded Theory typically builds theory inductively from the data without preconceived models, while SEM tests predefined, hypothesized models. We agree that combining these two approaches requires careful justification, and the transition from qualitative coding to quantitative model testing should be transparent. In response to your feedback, we have made the following clarifications and adjustments:

(1)Justification for Combining Grounded Theory and SEM: We use Grounded Theory in the initial phase of our study to inductively identify the key driving factors of sports tourism industry integration from the data. Grounded Theory is employed here to ensure that the driving factors are rooted in empirical data rather than being preconceived. Once these key factors are identified, we move to the next phase, where Structural Equation Modeling (SEM) is used to test the relationships between these identified factors and assess their impact on the industry integration process.

The rationale for using both methodologies is to ensure a comprehensive approach: Grounded Theory allows for theory development without pre-existing bias, and SEM provides a robust tool for quantitatively validating the relationships and testing the model derived from Grounded Theory. This hybrid approach is intended to strengthen the validity and reliability of our findings by combining both qualitative and quantitative perspectives.

(2) Clarifying the Transition: To address the lack of transparency in the transition between qualitative coding and quantitative model testing, we have clarified this process in the revised manuscript. Specifically:

In the methodology section, we now explain that Grounded Theory is first used to identify the driving factors through a three-stage coding process (open coding, axial coding, and selective coding). The final identified factors then form the basis of the hypothesized model for SEM analysis.

We have also added a section “Methodological justification” to describe how the relationships between these identified factors were mapped into a structural model for SEM, highlighting the logical progression from qualitative findings to quantitative hypothesis testing.

(3) Updated Methodology Section: The updated methodology section now clearly outlines the step-by-step process, from data collection and qualitative analysis (Grounded Theory) to the subsequent formulation of hypotheses and testing with SEM. This ensures that the overall methodological approach is logically consistent and transparent.

The above revisions can be found in the sections of “Materials and methods” in the text, with changes highlighted in red. We hope these revisions clarify the methodological approach and address your concerns about the consistency and transparency of the research design. Thank you for helping us improve the rigor and clarity of our study.

***Comment 5:***

*While the SEM model yields statistical significance for all six hypotheses, the impact of core constructs like market demand (β=0.065) and government behavior (β=0.052) is minimal.*

**Response 5:**

Thank you for your insightful comment regarding the minimal impact of constructs such as market demand (β=0.065) and government behavior (β=0.052). In response, we have added a detailed discussion in the Results and Discussion sections to clarify the potential reasons behind these findings. The following is a summary of our response and the key modifications we have implemented:

(1)We have added an in-depth discussion to explain the relatively low impact of market demand and government behavior. For market demand, we highlight that the sports tourism market in the Guangdong-Hong Kong-Macao Greater Bay Area (GBA) is still in its developmental stage, with rapidly evolving consumer preferences and demand patterns. This dynamic nature may dilute the direct impact of market demand on industry integration. Regarding government behavior, we acknowledge that while government policies provide essential infrastructure and policy support, their effectiveness at the micro-enterprise level can be limited by challenges in cross-regional coordination and implementation within the GBA.

(2)Emphasis on Broader Significance. We stress that despite their minimal direct impact, these factors play crucial roles in shaping the broader environment for sports tourism development. Government policies offer foundational support for industry growth, while market demand continues to push enterprises toward innovation and diversification.

(3)Propose future research directions. We suggest that future research further explore how to enhance the influence of these factors, such as by optimizing the policy implementation mechanism or accurately grasping changes in market demand.

The above revisions can be found in the sections of “Results” and “Discussion” in the text, with changes highlighted in red. We believe these revisions address your concerns and provide a more nuanced understanding of the factors influencing sports tourism industry integration in the GBA. Thank you again for your insightful comments.

***Comment 6:***

*Table 8 and 9 contain too much redundant data without corresponding synthesis in the main text. The lack of condensed insights or grouped patterns makes interpretation difficult and offers minimal value to readers.*

**Response 6:**

We sincerely thank the reviewer for this critical insight. We have implemented comprehensive revisions to enhance data conciseness and interpretive clarity:

(1)Table 9 (measurement model paths) has been removed entirely. Key metrics (factor loading ranges) are now integrated into Table 6 (Reliability & Validity).

Rationale: Detailed item-level coefficients (e.g., GA1-GA6) are peripheral to hypothesis testing; retaining ranges preserves validity evidence without redundancy.

(2)Table 8 is streamlined to essential hypothesis results, retaining only:

Hypothesis notation (H1-H6), Path relationships (e.g., GA→IE), Standardized coefficients (β), p-values and with significance markers. Deleted: Unstandardized coefficients, S.E. and C.R. values (redundant with β and p-values).

(3) New synthesis in Results explicitly interprets grouped patterns: Human capital (β = 0.619) and technological innovation (β = 0.541) dominate integration outcomes, while government behavior (β = 0.052) and resource environment (β = 0.041) show minimal effects—likely due to policy fragmentation across administrative boundaries.

This directly links Table 8 data to: (a) Driver hierarchy (strong/moderate/weak effects); (b) Contextual explanations (cross-regional governance challenges).

(4) The complete measurement item coefficients in the original Table 9 have been replaced in the supplementary data table to enhance transparency and avoid text overload.

These revisions have avoided excessive redundancy of data in the table while enhancing the integration of insights. We believe this significantly enhances reader engagement with core findings.

***Comment 7:***

*The manuscript contains repetitive phrasing, vague expressions ("significant support", "relatively weak effect", "key driver") and insufficient transitions between theoretical constructs and empirical results.*

**Response 7:**

Thank you for your valuable comments. In response to your concerns regarding the “repetitive phrasing, vague expressions (such as ‘significant support,’ ‘relatively weak effect,’ ‘key driver’), and insufficient transitions between theoretical constructs and empirical results,” we have made detailed revisions and hope the following changes fully address your concerns:

(1)Revisions to repetitive phrasing: We reviewed the manuscript for redundant expressions and have appropriately streamlined them. For example, expressions like “significant support” and “key driver” have been replaced with precise explanations based on concrete empirical findings (e.g., β values) and their corresponding theoretical backgrounds, thereby avoiding overly general wording.

(2)Clarification of vague expressions: For vague phrases such as “relatively weak effect,” we have quantified the magnitude of each influence using empirical data (e.g., β values). In the conclusions, we now describe the specific strength of each driver in detail, avoiding broad terms like “weak” or “strong.” For instance, the influences of government behavior, market demand, and resource environment are now clearly presented with numerical values (β values) and compared against the theoretical framework.

(3)Transitions between theoretical constructs and empirical results: We have strengthened the transitional language between theoretical analysis and empirical findings, explicitly highlighting the connection between theory and data. For example, each theoretical construct (such as government behavior, market demand, human capital, etc.) is now clearly supported by empirical results, and the conclusion section explicitly details how the theoretical analysis aligns with the empirical validation.

The above revisions can be found in the sections of “Abstract” “Results” and “Conclusions” in the text, with changes highlighted in red. Through these revisions, we have aimed to make the manuscript more concise and clear, while ensuring a smoother logical flow between the theoretical framework and empirical data.

***Comment 8:***

*The authors are encouraged to refine their theoretical framing, consider an alternative modeling strategy (e.g., PLS-SEM or system dynamics), and increase the scientific contribution before resubmitting.*

**Response 8:**

Thank you for your valuable feedback! In response to your suggestion to“refine the theoretical framing, consider an alternative modeling strategy (e.g., PLS-SEM or system dynamics), and increase the scientific contribution before resubmitting,” we have carefully considered your advice and made the following revisions. While we understand the advantages of methods like PLS-SEM and system dynamics in handling complex models or dynamic systems, after further discussion, we have decided to retain our current modeling approach. We believe that the existing method is well-suited to address the research questions of this study and handle the characteristics of our data. Here are the specific revisions and responses:

(1) Refining Theoretical Framing: In the theoretical framework section, we have further clarified the relationships between the variables. We explicitly defined each construct and elaborated on the causal relationships between them, strengthening the logical foundation of our hypotheses. In particular, we added more theoretical explanations regarding the interactions among variables like government behavior, market demand, and resource environment to ensure greater clarity and coherence in the theoretical framework.

(2) Explanation for Not Adopting Alternative Modeling Strategies: We have carefully considered the suggestion to use alternative modeling strategies such as PLS-SEM or system dynamics. However, after thorough deliberation, we maintain that the SEM approach is most suitable for our research objectives and data characteristics. SEM has significant advantages in confirmatory analysis, which can effectively evaluate the fit degree between theoretical models and data, and provide reliable results to support our research hypotheses. It also offers strong causal inference capabilities, which align well with our aim to explore the relationships between driving factors and integration outcomes.

(3) Emphasized Scientific Contributions: By integrating Grounded Theory with Structural Equation Modeling (SEM), we have identified the key drivers of sports tourism integration and proposed specific policies to boost high-quality regional economic development. These findings offer valuable insights for both the theoretical and practical aspects of the sports tourism industry.

We are committed to enhancing the quality of our manuscript and appreciate the guidance provided by your comments.

Sincerely,

The Authors
