# Supplementary material for: Drivers and dynamic mechanisms of sports tourism integration in cross-border regions: Evidence from the Guangdong-Hong Kong-Macao Greater Bay Area
Source: PLoS One. 2026 Mar 23;21(3):e0344124. doi: 10.1371/journal.pone.0344124 (PMC13008054; doi:10.1371/journal.pone.0344124)
Supplement: Attachment — Submitted filename: Peer REVIEW REPORT 2.docx [file pone.0344124.s004.docx]

**Peer Review Report**

**Manuscript Title**: Internet and Mobile Phone Use and Acceptance of Mobile Health Interventions Among Tuberculosis Patients in Cameroon

**Manuscript ID**: PONE-D-25-06940R1

**General Assessment**

This manuscript explores the accessibility and acceptance of mobile health (mHealth) interventions among tuberculosis (TB) patients in Cameroon. The topic is of high public health relevance, especially within resource-limited settings. The authors have revised the manuscript following previous feedback, and several improvements are evident. However, methodological clarity, data representation, and ethical documentation still require enhancement to meet PLOS ONE standards.

**Title and Abstract**

Strengths: The title accurately captures the study focus. The abstract succinctly presents objectives, methods, and findings.

Weaknesses: The abstract omits sampling details and lacks mention of ethical clearance.

Recommendations: Briefly mention sample size, sampling method, and ethical approval in the abstract.

**Introduction**

Strengths: Provides context on TB burden in Cameroon and potential of mHealth.

Weaknesses: Limited discussion of theoretical frameworks guiding technology acceptance (TAM, UTAUT).

Recommendations: Integrate brief reference to existing acceptance models to support rationale.

**Methods**

Strengths: States study design and population clearly.

Weaknesses: Does not provide specific sampling technique (e.g., convenience, random), inclusion/exclusion criteria, or sample size justification.

Recommendations: Clarify recruitment strategy, power analysis (if done), and data collection instrument validation.

**Results**

Strengths: Relevant findings presented on technology use and mHealth acceptance.

Weaknesses: Tables lack p-values for some comparisons, and some interpretations seem anecdotal.

Recommendations: Ensure all statistical comparisons are reported with relevant significance metrics and include measures of variability (e.g., SD, CI).

**Discussion**

Strengths: Discusses public health implications and aligns findings with existing literature.

Weaknesses: Needs a clearer link between findings and their practical implications for mHealth implementation.

Recommendations: Address potential biases (e.g., urban vs rural TB populations) and limitations of cross-sectional design.

**Conclusion**

Strengths: Summarizes findings appropriately.

Weaknesses: Overstates generalizability.

Recommendations: Temper conclusions and suggest avenues for future research, including longitudinal and interventional studies.

**Figures and Tables**

Strengths: Tables are clear and relevant.

Weaknesses: Lack of visual summary (e.g., bar charts or infographics).

Recommendations: Include a chart summarizing key usage or acceptance indicators for better visual communication.

**Data Availability and Ethical Statement**

Weaknesses: Ethical approval is mentioned in-text but needs clearer elaboration in the ethical declaration section.

Recommendations: Provide full IRB name, approval number, and confirm informed consent procedures in a dedicated section.

**References**

Strengths: Relevant and appropriately cited.

Weaknesses: Minimal mention of digital health frameworks.

Recommendations: Consider citing mHealth implementation literature in low-resource settings.

**Final Recommendation**

**Decision**: Minor Revision

The manuscript is promising but requires minor methodological and ethical clarifications before acceptance.
