# Supplementary material for: Drivers and dynamic mechanisms of sports tourism integration in cross-border regions: Evidence from the Guangdong-Hong Kong-Macao Greater Bay Area
Source: PLoS One. 2026 Mar 23;21(3):e0344124. doi: 10.1371/journal.pone.0344124 (PMC13008054; doi:10.1371/journal.pone.0344124)
Supplement: Attachment — Submitted filename: renamed_d0b62.docx [file pone.0344124.s005.docx]

****Response to Reviewers’ Comments****

We sincerely thank the editors and reviewers for their insightful comments and constructive suggestions, which have greatly improved the quality and clarity of our manuscript. We have carefully considered all the feedback and have revised the manuscript accordingly. Below, we provide a point-by-point response to the comments. All changes in the manuscript have been highlighted for ease of review.

**Comment 1:** Reference and Citation Format
The reference formatting is inconsistent. Please refer to the PLOS ONE sample manuscript or published articles in the journal to ensure proper citation style throughout.

**Response:**

We sincerely thank the reviewer for pointing out the inconsistencies in the reference formatting. We have thoroughly reviewed and revised the reference list and in-text citations throughout the manuscript to ensure full compliance with the PLOS ONE reference style, as illustrated in the journal’s sample manuscripts and author guidelines. Each entry has been carefully checked for consistency in author names, journal title abbreviations, punctuation, and the use of DOI links where applicable. We appreciate the reviewer’s attention to detail, which has significantly improved the professionalism and readability of the manuscript.

**Comment 2: Methodology - Sample Size Justification**

The methodology mentions that 16 respondents were interviewed. A clear justification for selecting this specific number is missing. I recommend increasing the number of interviewees to enhance the credibility and robustness of the findings.

**Response:**

We thank the reviewer for this valuable comment, which has helped us improve the methodological rigor of our manuscript. We have now provided a detailed justification for the sample size in the revised “Data collection” section (Lines 165–175).

In qualitative research guided by Grounded Theory methodology, sample size is not determined by statistical power calculations but by the principle of thematic saturation, the point at which new interviews cease to provide novel thematic insights or conceptual categories. Our iterative data collection and analysis process confirmed that after 16 interviews, core categories and themes began to repeat, indicating that saturation was achieved and a comprehensive understanding of the driving factors was attained.

Furthermore, to ensure the robustness and credibility of our findings within this sample size, we employed a purposive sampling strategy designed to capture maximum variation. Our 16 interviewees represent a diverse range of key stakeholders (e.g., senior managers, project planners, industry association leaders) from various types of organizations across all 11 cities in the GBA. This strategic selection ensures that the data is rich, comprehensive, and contextually grounded, which is the primary objective of qualitative inquiry.

We have added citations to methodological literature that support this approach. We believe this explanation clarifies that our sample size is methodologically sound and sufficient for the goals of our study.

**Comment 3: Presentation of Tables 3 and 4**

Tables 3 and 4 may be better presented as Appendix A and Appendix B, respectively, and placed after the references section at the end of the manuscript.

**Response:**

We thank the reviewer for this constructive suggestion. In accordance with the comment, we have moved the original Table 3 (Example of open coding) and Table 4 (Results of axial coding) to the Supporting Information section. They are now presented as:

S1 Table. Example of open coding

S2 Table. Results of axial coding

These tables have been removed from the main text and placed in the Supporting Information file after the References section, as recommended. This adjustment helps streamline the main manuscript while retaining essential methodological details for interested readers. We believe this presentation improves the readability of the paper.

**Comment 4: Research Questions and Conclusion**
The research questions, conclusions, and recommendations need to be reconsidered. The current outcomes appear overly generalized. Since the study context, participants, and approach are all unique, I strongly recommend that the findings reflect this uniqueness, highlighting the originality and alignment with the study’s scope as stated at the beginning.

**Response:**
We sincerely thank the reviewer for this valuable comment. We fully agree that the original research questions, conclusions and recommendations required refinement to better reflect the uniqueness of the Guangdong-Hong Kong-Macao Greater Bay Area (GBA) context, and to highlight the originality and alignment of the study with its stated scope. Accordingly, we have made substantial revisions to both the Introduction and the Conclusions and Recommendations sections:

1. Research Questions

The revised research questions now emphasize the specific cross-border context of the GBA, highlighting the distinct institutional systems, resource environments, and policy dynamics that drive the integration of the sports tourism industry. The questions have been reworded to better capture the dynamic and complex interactions among key drivers within the GBA region.

The first question has been updated to clarify the role of critical factors shaping the integration of the sports tourism industry, with a focus on the institutional and economic dynamics of the GBA. The second question now explicitly addresses how these factors interact and their varying impacts within the unique institutional and policy landscape of the GBA. The third question has been revised to focus on the context-specific policy and practical recommendations for advancing sustainable integration, drawing directly from the empirical analysis and aligning with the revised conclusions. These revisions provide a clearer, more focused framework for understanding the dynamics of sports tourism industry integration in the GBA. The updated questions reflect the empirical depth of our study and better situate the research within the context of cross-border governance and regional complexity.

2. Conclusions and recommendations

The revised conclusions emphasize that the dynamics of sports tourism integration are profoundly shaped by the cross-border governance structure under “one country, two systems”, socio-economic diversity, and institutional complexities of the GBA. We highlight the hierarchical structure of drivers (human capital and technological innovation > enterprise development needs > market demand, resource environment, and government behavior), and interpret their effects within the region’s distinctive institutional landscape. We stress the study’s originality in bridging micro-level dynamics (enterprise behaviors, talent mobility) with macro-level frameworks (policy, institutional coordination), offering a context-sensitive and empirically grounded explanation of integration mechanisms.

The revised conclusion also explicitly links findings to the 15th National Games, positioning it as a strategic opportunity for implementing policy, digital, and talent-related initiatives. Finally, we clarify the theoretical and practical contributions: the study not only advances understanding of cross-border tourism integration but also provides timely insights for strengthening the GBA’s role as a globally competitive and innovation-driven metropolitan region.

The revised recommendations are now directly aligned with the empirical driver hierarchy, ensuring coherence between findings and policy implications. We avoid generalized prescriptions by tailoring recommendations to the unique cross-border challenges of the GBA, including policy fragmentation, resource allocation inefficiencies, and segmented market demand.

New emphasis is placed on (1) enhancing policy coordination across Guangdong, Hong Kong, and Macao; (2) building a digital integration hub powered by AI, big data, and cloud computing; (3) strengthening enterprise-led cross-border clusters and PPP models; (4) facilitating talent exchange platforms across the three jurisdictions; and (5) developing segmented markets and joint branding strategies, with the 15th National Games as a practical catalyst. These revisions make the recommendations firmly grounded in the GBA’s institutional and socio-economic context, while also offering transferable insights for other cross-border regions.

**Changes in manuscript:**

The revised Research Questions section is presented in lines 94–107.

The revised Conclusions section is presented in lines 422–449.

The revised Recommendations section is presented in lines 451–488.
